# Editorial

**Published:** 1872-09

**Authors:** 


### EDITORIAL.

### The Horace Wells Testimonial Fund.

We commend to the attention and earnest consideration of our readers the following appeal; and trust that for the accomplishment of its object it will receive the hearty co-operation and material aid of every member of the dental profession.

This work has been by far too long delayed; but it is gratifying to know that their is, even at this late day, an awakening sense of justice.

This testimonial ought to be secured without any very great effort; it is comparatively a small thing for each to do, and it should be promptly done.

It should not be necessary to keep importuning and urging the profession for a year or two. Twenty thousand dollars should be at once sent to the treasurer, and then quietly placed in the hands of Dr. Well's heirs. Acts of this character make men grow, and in the end would make some men more willing to die.

APPEAL IN BEHALF OF THE WELLS NATIONAL TESTIMONIAL FUND.

"It is proposed to raise a fund for the benefit of the family of the late Horace Wells, the discoverer of the principle of Anæsthesia, or the prevention of pain in surgical and dental operations, by the inhalation of nitrous oxide gas and other agents.

"Dr. Wells was a benefactor to the world.

He conferred upon it the greatest physical boon, in the prevention of pain in severe operations in general surgery and obstetrics, as well as in minor operations in dentistry. Without its aid, many of them would be impossible, while with it they have not only been rendered possible, but, directly and indirectly, tens of thousands of lives, as well as an untold amount of mental and physical anguish, have been saved. To accomplish this his own health, life and fortune were sacrificed in his zeal for the perfection of his great discovery, and his early death left those whom he loved and cherished unprovided with even the most moderate means of support.

"This appeal is therefore made, in behalf of *his family*, to the members of the dental profession, as well as to private citizens, benefited directly or indirectly by his great discovery, to aid in making up a Testimonial Fund as an act of tardy justice to the memory of an American citizen who deserves to rank with Morse and Fulton among the world's benefactors, and whose claim to everlasting and grateful remembrance is enhanced by the fact that *he loved his fellow-man better than himself.*

"We would, therefore, urge the dental profession to embrace this opportunity, and generously show their appreciation of Horace Wells and his discovery, and of the immense benefit it has been to them, by contributing to this fund.

"Subscriptions may be sent direct to the Treasurer of the Fund to whom all contributions will be paid over.

"Louis Jack, D. D. S., Daniel Neal, D. D. S., George T. Barker,

D. D. S., George T. Moffat, M. D., N. W. Hawes, M. D., Thomas B. Hitchcock, M. D., William H. Allen, Frank Abbott. M. D., J. H. McQuillen, D. D. S., E. Wildman, D. D. S., D. D. Smith, D. D. S., Thomas H. Chandler, M. D., Jacob L. Williams, M. D., J. S. Latimer, D. D. S., Norman W. Kingsley, D. D. S.

" *Treasurer*, Samuel S. White.

" *Secretary* (Dental Profession), James Truman, D. D. S., 1221 Spruce Street, Philadelphia."

### Difficulties in Dental Practice.

Among the casualities that occur to filled teeth there are none more frequent or more fatal in their results than the recurrence of decay in that part of the cavity, on the proximate parts of the teeth, next to the margin of the gum, or as some would express it, in the cervical wall of the cavity. Formerly it more often took place than now.

This is a very vulnerable point for the attack of decay: first, because the locality is one that affords ready lodgment, and retention of foreign substances in contact with the tooth, from which the brush and the ordinary means employed for cleansing the teeth will not remove it.

The teeth on the proximate sides are not so well covered and protected by enamel as upon other parts.

Another occasion of decay in the part referred to is found in the imperfect manner in which the operation of filling these cavities is accomplished. It is often quite difficult to make thorough excavation of the cervical walls of proximate cavities; and especially is this true so far as giving proper form is concerned. And when this part of the work has been well done there may be failure to properly adapt and consolidate the filling; or the margin of the cavity is fractured or injured to a greater or less extent by improper contact with the instrument. But none of these unfavorable circumstances may exist and yet decay occur at the cervical wall, and particularly if thorough cleanliness is not observed, but even this will sometimes fail to secure immunity from disease. Why is it that decay will sometimes under the most favorable circumstances take place.

There is perhaps no agent with which the teeth are brought into contact more definitely, and certainly injurious, than the vitiated mucus, the product of diseased mucous membrane. Inflammation of this tissue causes such a change in its secretion, that it will produce irritation and often decomposition of the dentine.

How important then that this tissue should be kept in a healthy condition, and every thing avoided that would produce a diseased or even an abnormal condition. Of the various ways in which the mucous membrane may be effected injuriously, there is perhaps none more common than the use of dental plates, badly constructed, or made of improper material; but with this fact the dental profession is comparatively well acquainted. There is, however, an almost entire overlooking, or ignoring, practically, of the fact that the mucous membrane covering the margin of the gums, and particularly that part between the teeth, is exposed to more and greater irritants than at any other locality. The margins of the gums have not the same support as the mucous membrane and its subjacent tissue elsewhere; and is more exposed to brusing and laceration by violent contact.

This pernicious condition so often found between the teeth, is produced in various ways. The gums by having foreign substances crouded against and pressed upon them will become diseased; or by overlapping the sharp and rough borders of cavities of decay the same result will be produced. Protruding and overlapping filling will usually produce a similar difficulty; unskillful and rough manipulations often produce disease: violent wedging causing bruising of the gum, forcing down ligatures or rubber to produce laceration should be avoided. Indeed it should be remembered that whatever will produce irritation of the margins of the gums is productive of injury to the teeth to a greater or less extent. The dentist should possess the most thorough knowledge of the structure of the mucous membrane, of its functions, and of the abnormal conditions and diseases to which it is liable; and the proper method of treatment for each.

### Screws.

We have been using Mack's screws with his instruments for their introduction, for some months past, and like them very much indeed. They supply a want, in certain cases, that was very definitely felt before their introduction.

Their use gives a far greater security to a certain class of operations than was ever attainable before. and that too with less risk of producing unfavorable results.

No dentist who desires to have at his command all the best things—which will enable him to best meet the difficult cases that often occur—can afford to be without these screws and the instruments for their use.

### The Ohio Dental College.

The opening of the twenty-seventh annual session of this institution will take place October 16th, when we hope all those who are to constitute the classes will be present so far as possible. The facilities were, we think, never before so good for giving a thorough course of training, as now; collegiate instruction has become a necessity for those who desire to enter the profession with usefulness, honor and assurance of success. The spirit of the times requires that every thing possible should be done for development and progress.

